# Lysophosphatidylcholine acyltransferase level predicts the severity and prognosis of patients with community-acquired pneumonia: a prospective multicenter study

**DOI:** 10.3389/fimmu.2023.1295353

**Published:** 2024-01-08

**Authors:** Li Chen, Jianbo Xue, Lili Zhao, Yukun He, Shining Fu, Xinqian Ma, Wenyi Yu, Yanfen Tang, Yu Wang, Zhancheng Gao

**Affiliations:** ^1^ Department of Respiratory, Beijing Ditan Hospital, Capital Medical University, Beijing, China; ^2^ Department of Respiratory & Critical Care Medicine, Peking University People’s Hospital, Beijing, China; ^3^ Department of Respiratory and Critical Care Medicine, Beijing Jishuitan Hospital, The Fourth Medical College of Peking University, Beijing, China

**Keywords:** lysophosphatidylcholine acyltransferase, mortality, community-acquired pneumonia, predictive value, etiology

## Abstract

**Background:**

Identifying the diagnosis as well as prognosis for patients presented with community-acquired pneumonia (CAP) remains challenging. We aimed to identify the role of lysophosphatidylcholine acyl-transferase (LPCAT) for CAP along with assessing this protein’s effectiveness as a biomarker for severity of disease and mortality.

**Methods:**

Prospective multicenter research study was carried out among hospitalized patients. A total of 299 CAP patients (including 97 severe CAP patients [SCAP]) and 20 healthy controls (HC) were included. A quantitative enzyme-linked immunosorbent test kit was employed for detecting the LPCAT level in plasma. We developed a deep-learning-based binary classification (SCAP or non-severe CAP [NSCAP]) model to process LPCAT levels and other laboratory test results.

**Results:**

The level of LPCAT in patients with SCAP and death outcome was significantly higher than that in other patients. LPCAT showed the highest predictive value for SCAP. LPCAT was able to predict 30-day mortality among CAP patients, combining LPCAT values with PSI scores or CURB-65 further enhance mortality prediction accuracy.

**Conclusion:**

The on admission level of LPCAT found significantly raised among SCAP patients and strongly predicted SCAP patients but with no correlation to etiology. Combining the LPCAT value with CURB-65 or PSI improved the 30-day mortality forecast significantly.

**Trial registration:**

NCT03093220 Registered on March 28th, 2017.

## Background

Community-acquired pneumonia (CAP), regarded highly prevalent globally, commonly resulted in infectious disease-related mortality in the United States (1, 2). Despite advances in antibiotics and other supportive treatments, some patients rapidly progress to severe pneumonia with fever and hypoxemia, and eventually could die due to acute respiratory syndrome or septic shock. The overall mortality is approximately 4–18% among patients admitted to hospital and around 50% among severe CAP (SCAP) presented cases (3, 4). Therefore, early identification of SCAP is important. Severe pneumonia is defined by meeting one primary or three secondary criteria (5, 6). Furthermore, the CURB-65 scoring systems and pneumonia severity index (PSI) are broadly used to grade CAP severity in clinical practice (7). Even when evaluated by PSI or CURB-65 scores, a delay in diagnosis cannot be completely avoided because both methods are mainly based on limited medical information. Delayed intensive care owing to delayed diagnosis is closely related to increased mortality. Biomarkers in the peripheral blood, evaluated by a simple blood test, can objectively indicate a specific diagnosis and provide prognostic information.

Lysophosphatidylcholine acyltransferase (LPCAT) is a lipid-modifying intracellular enzyme in many tissues, including alveolar type II cells in the lung (8), hepatocytes (9, 10), and red blood cells (11). LPCAT converts lysophosphatidylcholine to phosphatidylcholine in the presence of acyl-CoA (12), a step in Land’s cycle (13). It is involved in lipid metabolism and the maintenance of membrane integrity. The progression and metastasis of many diseases, including multiple cancers, are related to the overexpression of the LPCAT gene or the LPCAT level in the body (14–16). In the lung tissue, phosphatidylcholine from the LPCAT remodeling pathway is converted into dipalmitoylphosphatidylcholine, which is responsible for the surface tension-lowering properties of the surfactant via the remodeling pathway of dipalmitoylphosphatidylcholine synthesis (8).

Recently, artificial intelligence, represented by deep learning, has been increasingly applied in clinical practice. From DNA sequence analysis to medical image processing, the application of these emerging technologies can help clinicians not only ease clinical procedures but also understand the features of the disease. Demographic and clinical data have been broadly used for psychological illness prediction and identification using deep learning methods (17, 18). In respiratory disease assessment, convolutional neural networks are typically applied to medical image processing (chest radiography or computed tomography scan) (19). The Food and Drug Administration in America has granted regulatory approval for the deep learning diagnostic software used in clinical practice (20).

In this research, we assumed that the severity of CAP was related to the level of LPCAT in peripheral blood. We measured the LPCAT level by an ELISA (enzyme-linked immunosorbent assay) for determining the LPCAT role in SCAP and evaluating the predictive values for LPCAT as disease severity and prognosis among CAP patients in the early stage. We also applied a deep learning algorithm to process laboratory test results to distinguish patients with CAP into SCAP and NSCAP.

## Methods

### Study population

This prospective, observational and multi-center study was conducted among hospitalized patients between January 2017 and October 2018. All the samples were obtained from Peking University People’s Hospital (PKUPH), Tianjin Medical University General Hospital, Wuhan University People’s Hospital and Fujian Provincial Hospital. The study was approved by the Institutional Review Board of PKUPH (No. 2016PHB202-01) and registered at ClinicalTrials.gov (ClinicalTrials.gov ID, NCT03093220). Informed consent in written was obtained from all participants. All cases enrolled for the research were CAP upon diagnosis and already hospitalized either at the respiratory or intensive care unit. CAP was defined by the following criteria (4): (1) a chest radiograph showing either a new patchy infiltrate, leaf or segment consolidation, ground glass opacity, or interstitial change; (2) at least one of the following signs – (a) the presence of cough, sputum production, and dyspnoea; (b) core body temperature >38.0°C; (c) auscultatory findings of abnormal breath sounds and rales; or (d) peripheral white blood cell counts >10 × 10^9^/L or<4 × 10^9^/L; and (3) symptom onset that began in the community, rather than in a healthcare setting. SCAP was diagnosed by the presence of at least one major criterion, or at least three minor criteria, as follows (6). Major criteria: (1) requirement for invasive mechanical ventilation and (2) occurrence of septic shock with the need for vasopressors. Minor criteria: (1) respiratory rate ≥30 breaths/min; (2) oxygenation index (PaO_2_/FiO_2_) ≤250; (3) presence of multilobar infiltrates; (4) presence of confusion; (5) serum urea nitrogen ≥20 mg/dL; (6) white blood cell count ≤4 × 10^9^/L; (7) blood platelet count<100 × 10^9^/L; (8) core body temperature<36.0°C; and (9) hypotension requiring aggressive fluid resuscitation. The exclusion criteria were age<18 years, or the presence of any of the following: pregnancy, immunosuppressive condition, malignant tumor, end-stage renal or liver disease, active tuberculosis, or pulmonary interstitial fibrosis. We also recruited 20 sex and age matched healthy volunteers to set up a baseline LPCAT level in healthy individuals. Assessing LPCAT level as predictor for SCAP or NSCAP is considered as primarily an endpoint for this research. The secondary endpoint was the mortality of 30 days after CAP onset. Outcomes were assessed at hospital discharge and at 30 days following inclusion in the study using structured telephone interviews.

### Sample size calculations

We assumed that the mortality rates of NSCAP (P_0_) and SCAP (P_1_) were 0.05% and 0.25%, respectively (6, 21). An alpha level of 0.05 at 95% confidence of interval was considered significant to encounter type I error. A level of 0.01 set for type II (β) error because these parameter settings can provide a power of 90% for the study. The test standard deviations were Z_α_=1.96 and Z_β_=1.282. The sample size was calculated below.


$$R=\frac{P_{1}}{P_{0}}$$

$$A=\;P_{1}\left(1-P_{0}\right)+P_{0}\left(1-P_{1}\right)$$

$$B=\left(R-1\right)P_{0}\left(1-P_{0}\right)$$

$$K=\left(A+B\right)\left(RA-B\right)-R{\left(P_{1}-P_{0}\right)}^{2}$$

$$N_{NSCAP}^{\prime }=\frac{Z_{\beta }^{2}K+\;Z_{\alpha }^{2}(A+B{)}^{2}+2Z_{\alpha }Z_{\beta }(A+B)\sqrt{K}}{{\left(P_{1}-P_{0}\right)}^{2}\left(A+B\right)}=121$$

$$N_{SCAP}^{\prime }=\frac{N_{NSCAP}^{\prime }}{R}=25$$

We assumed that there would be a 10–30% ineligible inclusion. We then calculated the primary numbers of cases diagnosed with NSCAP and SCAP.


$$N_{NSCAP}=\frac{N_{NSCAP}^{\prime }}{1-30\%}=173$$

$$N_{SCAP}=\frac{N_{SCAP}^{\prime }}{1-30\%}=36$$

### Microbiological evaluation

Lower respiratory tract specimens including sputum, endotracheal aspiration was collected during the first 24 hours after hospital admission. Bronchoalveolar lavage (BAL) samples were obtained whenever possible within 7 days after admission. The specimens were stored in sterile sample tubes, deep-frozen at −80°C until analyzed in a central laboratory at Peking University People’s Hospital. All samples were analyzed with multiplex real-time PCR assays for viral detection and DNA-based quantitative loop-mediated isothermal amplification (qLAMP) assays for bacterial detection.

Total viral nucleic acids were extracted from respiratory samples using a QIAamp MinElute Virus Spin Kit (Qiagen Inc., Valencia, CA, USA). The presence of common respiratory pathogens were screened using an AgPath-ID™ One-Step real-time polymerase chain reaction (RT-PCR) kit (Ambion) with the FTD respiratory pathogens 21 kit (Fast Track Diagnosis, Luxembourg), which included influenza A/B, influenza A H1N1, rhinovirus, coronaviruses (NL63, 229E, OC43, and HKU1), parainfluenza viruses, human metapneumovirus A/B, bocavirus, respiratory syncytial virus A/B, adenovirus, parechovirus, and enterovirus. A virus was considered as the etiology of CAP when the Ct value was<30, using GAPDH as an internal control (22).

Loop-mediated isothermal amplification (LAMP) assays were used to detect 13 common bacterial pathogens of CAP, including Streptococcus pneumoniae, Staphylococcus aureus, Methicillin-resistant Staphylococcus aureus, Escherichia coli, Klebsiella pneumoniae, Pseudomonas aeruginosa, Acinetobacter Baumannii, Stenotrophomonas maltophilia, Haemophilus influenza, Legionella pneumophila, Mycobacterium tuberculosis, Mycoplasma pnedumoniae, and Chlamydia pneumoniae. In our previous studies, LAMP assay had been proved to be an effective technique for detection of bacteria and atypical pathogens (23). A bacterium was considered to be the causative pathogen only if the DNA concentration was over 10^4^ copies/mL. If no pathogenic bacteria, viruses or atypical pathogens were detected in the sample, it is defined as “unknown”.

### Data collection

The clinical characteristics of participants were evaluated and recorded by attending physicians at admission. Peripheral venous blood was sampled within 8h after hospitalization, the plasma was immediately separated by *in-situ* centrifugation, transported in a dry ice environment, and stored at -80°C for subsequent analysis. Moreover, laboratory examination and chest imaging were performed within 24 h in situ. Scores for PSI and CURB-65 were calculated from clinically data.

### Measurement of LPCAT level

We measured plasma LPCAT level by using a quantitative enzyme-linked immunosorbent assay kits (Shanghai Enzyme-linked Biotech, Shanghai, China) in duplicate. For examination the details from manufacturer’s instructions were followed. The inter- and intra-assay coefficients of variation were< 15% and 10%. The quantities of plasma LPCAT were measured by a standard curve using CurvExpert Professional 2.6.3 (Hyams Development, Madison, WI, USA).

### Dataset creation, model development and performance evaluation

We created the dataset, the parameters of which are listed in **Supplementary Data Sheet 1**. We applied a deep-learning method, multilayer perceptron (MLP), to build a joint prediction model for the SCAP with multiple indicators. Eight dimensions representing the eight laboratory test results were passed to a dense layer with eight units as the input layer. The variables from the input layer were then passed to several hidden layers with different numbers of hidden units. We attempted several combinations of the hidden layers. The number of hidden layers was changed from 3 to 10 (including the input and output layers). The hidden units in each layer were also changed from 8 to 128. Finally, we applied a 10-layer multilayer perceptron with different hidden units to each layer. We also applied L2-norm regularization to the output layer to reduce overfitting. Further details regarding the MLP structure are presented in **Table 1**. We chose Adam (24), which has an automatic learning rate, as the optimizer. The model was trained for up to 500 epochs with a batch size of 16. Hyperparameters were adjusted based on a 5-fold cross-validation of the entire training dataset.

**Table 1:** Multilayer perceptron structure of prediction model.

Layer Name	Input	Output	Activation Function	Regularization	Kernel Initializer
Input Layer	8	128	Sigmoid	None	He Normal
Dense Layer 1	128	128	Sigmoid	None	He Normal
Dense Layer 2	128	64	Sigmoid	None	He Normal
Dense Layer 3	64	64	Sigmoid	None	He Normal
Dense Layer 4	64	32	Sigmoid	None	He Normal
Dense Layer 5	32	32	Sigmoid	None	He Normal
Dense Layer 6	32	16	Sigmoid	None	He Normal
Dense Layer 7	16	8	Sigmoid	None	He Normal
Dense Layer 8	8	2	Sigmoid	None	He Normal
Output Layer	2	2	Sigmoid	L2-norm	He Normal

To evaluate the model performance, we performed prediction on the test dataset features and applied the confusion matrix and ROC curve by comparing the labels in the test dataset. Sensitivity, specificity, and Youden index were calculated based on the confusion matrix. The AUC was also calculated using a ROC curve analysis. To evaluate the contribution of each input variable, we applied the dropout method by creating a series of dropout datasets. The details of the dropout dataset are elaborated in the section “Dataset Creation.” When performing an SCAP prediction on a drop-out dataset, a significant drop in the Youden index or AUC suggested a significant degradation in the model prediction without specific variables in the drop-out dataset, which also suggested the importance of this dropout variable. Model training and prediction were performed using the open-source platform TensorFlow (v2.8, Google Inc. USA) (25). The confusion matrices and AUCs were calculated using the Python package scikit-learn (v1.1.2), and the ROC curves were plotted using the Python package matplotlib (v3.5.3 The Matplotlib Development Team).

### Statistical analysis

The mean ± SE of the mean was used to present continuous variable with normal distribution whereas median (interquartile range) was used for non-normally distributed continuous variables. All the categorical variables are expressed as count or numbers (percentages). Student’s t-tests and Mann–Whitney U tests were employed for comparing both groups for normal distribution of data with homogeneity of variance and others, respectively. One-way ANOVA (analysis of variance) along with Kruskal-Wallis comparison test was employed for multiple groups comparison. Correlations between variables were assessed using Pearson’s correlation test or Spearman’s rho test. The receiver operating characteristic (ROC) curves, areas under the curve (AUCs), optimal threshold values, sensitivity, and specificity were calculated to compare the predicted values of different variable combinations. The 30-day survival curve was established using Kaplan-Meier method. GraphPad Prism version 6.01 (GraphPad Software, La Jolla, California, USA) and MedCalc statistical software version 15.2.2 (MedCalc Software, Ostend, Belgium) were used for all statistical analyses. Confidence intervals (CIs) were established at 95% in this study, and a two-sided p-value of 0.05 was considered statistically significant.

## Results

### Patient characteristics

299 patients with CAP were enrolled in this research, of whom 97 were diagnosed with SCAP. Twenty patients (20.6%) died during their hospitalization within 30 days of admission, due to CAP or its complications, including septic shock or multiple organ dysfunction syndromes. The remaining 279 patients recovered either discharged or were shifted within 30 days to the intensive care unit. white blood cell (WBC) count, C-reactive protein (CRP), bilateral change, pleural effusion on chest imaging, and pathogen detection showed significant differences among patients presented with NSCAP and SCAP. The demographic along with clinical characteristics of the study patients are presented in **Table 2**.

**Table 2:** Demographic and clinical characteristics of the 299 participants with CAP enrolled in this study.

Characteristic	non-SCAP (N=202)	SCAP (N=97)	*p* value
Male sex — no. (%)	114 (56.44)	55 (56.70)	0.361
Age — years	57.50 (45.00-69.00)	57.90 ± 16.46	0.570
BMI	21.24 (19.19-23.67)	22.49 (21.26-22.84)	<0.0001
Smoking history — no. (%)	34 (16.83)	16 (16.50)	0.542
Underlying diseases— no. (%)			
Chronic heart failure	11(5.45)	5 (5.20)	0.578
Diabetes mellitus	34 (16.83)	14 (14.43)	0.364
Cerebrovascular disease	14 (6.93)	18 (18.56)	0.003
Chronic liver disease	5 2.48)	7 (7.22)	0.054
Chronic renal disease	0 (0)	3 (3.09)	0.033
Bronchiectasis	1 (0.50)	3 (3.09)	0.102
Chronic obstructive pulmonary disease	6 (2.97)	9 (9.28)	0.023
Physical examination			
T max (°C)	39.85 ± 1.61	39.10 ± 1.09	0.590
Respiratory frequency (times/min)	22 ± 1	27 ± 4	<0.0001
Heart rate	89 ± 7	100 ± 23	<0.0001
Blood oxygen saturation	97.00 ± 1.41	91.37 ± 14.01	0.003
Laboratory results			
WBC (×10^9^/L)	8.20 (6.03-10.48)	7.60 (5.80-18.35)	<0.0001
NEU (%)	79.50 (67.75-84.20)	85.50 (77.90-88.80)	<0.0001
LYM (%)	12.10 (7.95-24.50)	7.70 (3.20-15.40)	<0.0001
NLR	4.08 (2.50-7.37)	11.53 (5.04-26.84)	<0.0001
PLT	225.75 ± 100.26	238.53 ± 97.79	<0.0001
ALT	36.25 (25.08-89.73)	34.00 (20.00-94.00)	<0.0001
AST	58.25 ± 49.43	53.59 ± 31.03	0.001
BUN	3.57 ± 1.62	5.33 ± 2.55	<0.0001
Scr	84.5 (57.5-111.5)	59.00 (42.00-61.00)	0.433
ALB	34.00 (24.80-40.05)	30.00 (28.00-32.00)	<0.0001
ESR	48.00 ± 43.03	64.13 ± 26.56	0.012
CRP	76.53 ± 75.28	89.32 ± 95.25	0.344
PCT	0.18 (0.06-2.40)	1.33 (0.47-1.71)	0.003
PaO_2_	91.55 (78.30-104.43)	68.90 (59.20-122.00)	<0.0001
PaO_2_/FiO_2_	435.5 ± 68.48	248.93 ± 101.93	0.526
PaCO_2_	29.43 ± 3.77	48.05 ± 42.09	0.555
SaO_2_	97.28 ± 1.35	91.63 ± 8.44	0.013
HCO_3_	20.80 (19.03-25.28)	23.70 (22.80-26.00)	0.054
Chest X-ray			
Bilateral lung infection— no. (%)	60 (29.70)	83 (85.60)	<0.0001
Pleural effusion— no. (%)	16 (7.90)	31 (31.96)	<0.0001
Detected pathogen— no. (%)			
Virus	22 (10.90)	19 (19.60)	0.033
Bacteria	34 (16.80)	24 (24.70)	0.073
Atypical pathogen	27 (13.40)	1 (1.00)	<0.0001
Mixed pathogen	24 (11.90)	10 (10.3)	0.425
Unknown	98 (48.50)	44 (45.40)	0.349
CURB-65			
Score points	0 (0-1)	1 (0-2)	<0.0001
0	130	20	
1	52	28	
2	18	29	
3	2	14	
4	0	6	
PSI			
Score points	57.00 ± 26.00	88.00 ± 37.00	<0.0001
≤ 70	134	21	
71-90	49	18	
≥ 91	19	59	
**30-day mortality-no. (%)**	0 (0.00)	20 (20.60)	<0.0001

Descriptive statistics. Variables are expressed as numbers (percentages). Continuous variables are expressed as the mean ± standard deviation (mean ± SD) when they met the normal distribution, and continuous nonparametric data are presented as the median and interquartile ranges (25^th^ and 75^th^ percentiles).

### LPCAT level in each group

**Figure 1:**
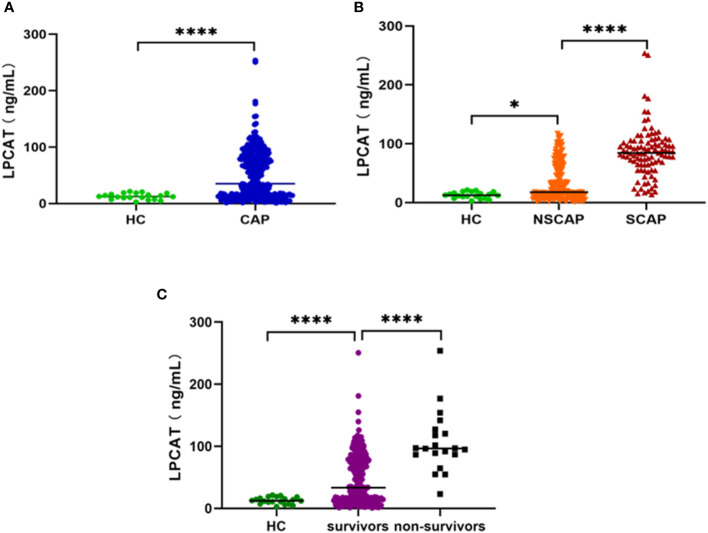
Plasma LPCAT levels in different groups. **(A)** plasma LPCAT levels in healthy control and CAP patients. **(B)** plasma LPCAT levels in healthy control and patients with NSCAP or SCAP. **(C)** plasma LPCAT levels in healthy control and CAP patients survived or didn’t survive. * p<0.05; **** p<0.0001.

The plasma LPCAT level in the 20 healthy volunteers was 12.71 ± 5.40 ng/mL. The level of plasma LPCAT at admission was 35.13 (13.88-83.03) ng/mL among CAP patients, found to be greater than that in healthy individuals (p< 0.05, **Figure 1A**). The plasma LPCAT level was significantly higher among SCAP patients comparison to those NSCAP (p-value<0.05, **Figure 1B**). For those who died within 30 days after diagnosis, the plasma LPCAT level was also significantly higher than that of survivors (p-value< 0.05, **Figure 1C**).

**Figure 2:**
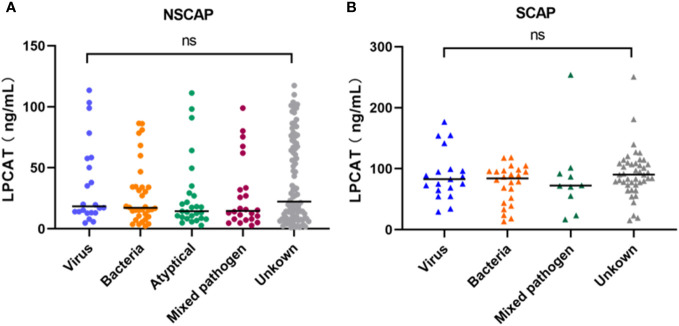
Plasma LPCAT levels of NSCAP patients **(A)** and SCAP patients **(B)** with different pathogens. ns, not-statistically significant.

The CAP etiology was categorized into the following pathogen groups: bacterial, viruses, atypical pathogens (Chlamydia pneumoniae, Mycoplasma pneumoniae, and Legionella pneumophila), mixed pathogens, and unknown pathogen group. Regardless of the NSCAP or SCAP status, no differences were observed in LPCAT levels among CAP patients presented different CAP etiological variations (p-value > 0.05, **Figure 2**).

### Correlation between plasma LPCAT level and CAP severity

**Figure 3:**
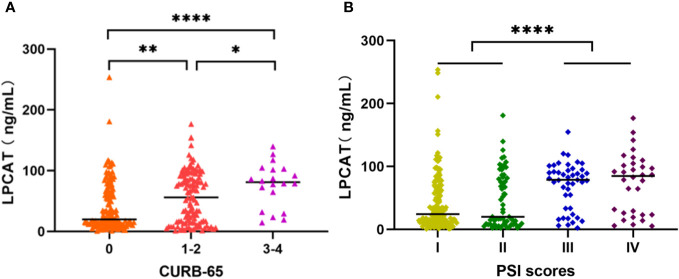
Plasma LPCAT levels in patients with CAP across different severity, divided by CURB-65 **(A)** and PSI scores **(B)**. * p<0.05; ** p<0.01; **** p<0.0001.

**Figure 4:**
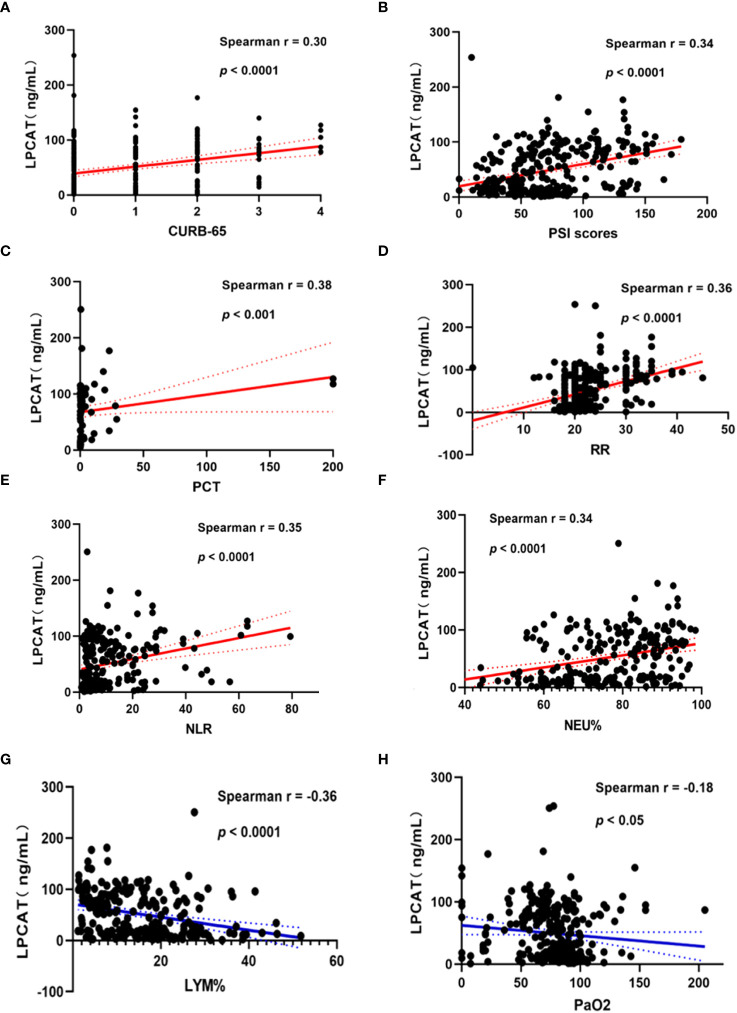
Correlation of plasma LPCAT levels with different indicators of CAP patients’ severity representing by CURB-65 **(A)** and PSI scores **(B)** or other examination results including PCT **(C)**, respiratory rate **(D)**, NLR **(E)**, NEU% **(F)**, LYM% **(G)**, PaO_2_ **(H)**.

We chose the PSI and CURB-65 scoring systems for evaluation the CAP severity among patients. The results found that levels of LPCAT in CURB-65 high score group were significantly greater than low score groups (**Figure 3A**). Similarly, the LPCAT levels of patients with CAP with PSI grades III or IV were significantly greater than PSI grades I or II patients (**Figure 3B**). The plasma LPCAT level at admission showed positive correlation with both of CURB-65 and PSI scores (**Figures 4A, B**, Spearman = 0.30 and 0.34, respectively; p-value< 0.0001). LPCAT level in CAP patients also showed positive correlation with the procalcitonin (PCT) level (Spearman = 0.38, p-value< 0.0001), respiratory rate (Spearman = 0.36, p-value< 0.0001), neutrophil/lymphocyte ratio (NLR) (Spearman = 0.35, p-value< 0.0001), and neutrophil percentage (NE%) (Spearman = 0.34, p-value< 0.0001) (**Figures 4C–F**). The plasma LPCAT level in patients negatively correlated with lymphocyte percentage (LY%) and PaO_2_ (**Figures 4G, H**, Spearman = -0.36 and -0.18, respectively; p< 0.0001).

### Severity prediction value of LPCAT in patients with CAP

As shown in **Table 3**, LPCAT showed the highest predictive value for SCAP compared with the other blood test results or CURB-65/PSI score. With 51.36 ng/mL optimal cut-off, AUC of LPCAT in predicting SCAP reached 0.853. The AUCs for CURB-65 and PSI were 0.773 (0.720-0.820) and 0.823 (0.774-0.865), respectively.

**Table 3:** Areas under the curve (AUCs) and thresholds for predicting SCAP in CAP patients.

	AUC	95% CI		Sensitivity	Specificity	Threshold	*p* value
		Lower limit	Higher limit				
LPCAT1	0.853	0.808	0.891	84.54%	72.77%	>51.36	<0.0001
WBC	0.640	0.582	0.696	58.70%	66.67%	>9.47	0.0001
NEU %	0.739	0.677	0.796	75.00%	68.15%	>78.00	<0.0001
LYM %	0.757	0.693	0.814	63.95%	79.34%	≤ 9.72	<0.0001
NLR	0.764	0.700	0.820	72.09%	71.07%	> 6.48	<0.0001
ESR	0.656	0.576	0.730	72.58%	65.59%	> 47.00	0.0005
CRP	0.536	0.453	0.617	45.76%	72.83%	> 105	0.4735
PCT	0.701	0.592	0.795	71.93%	68.97%	> 0.313	0.0008
CURB-65	0.773	0.720	0.820	78.02%	65.13%	> 0	<0.0001
PSI	0.823	0.774	0.865	64.84%	88.72%	> 86	<0.0001

### Performance of the MLP-developed SCAP prediction model

**Figure 5:**
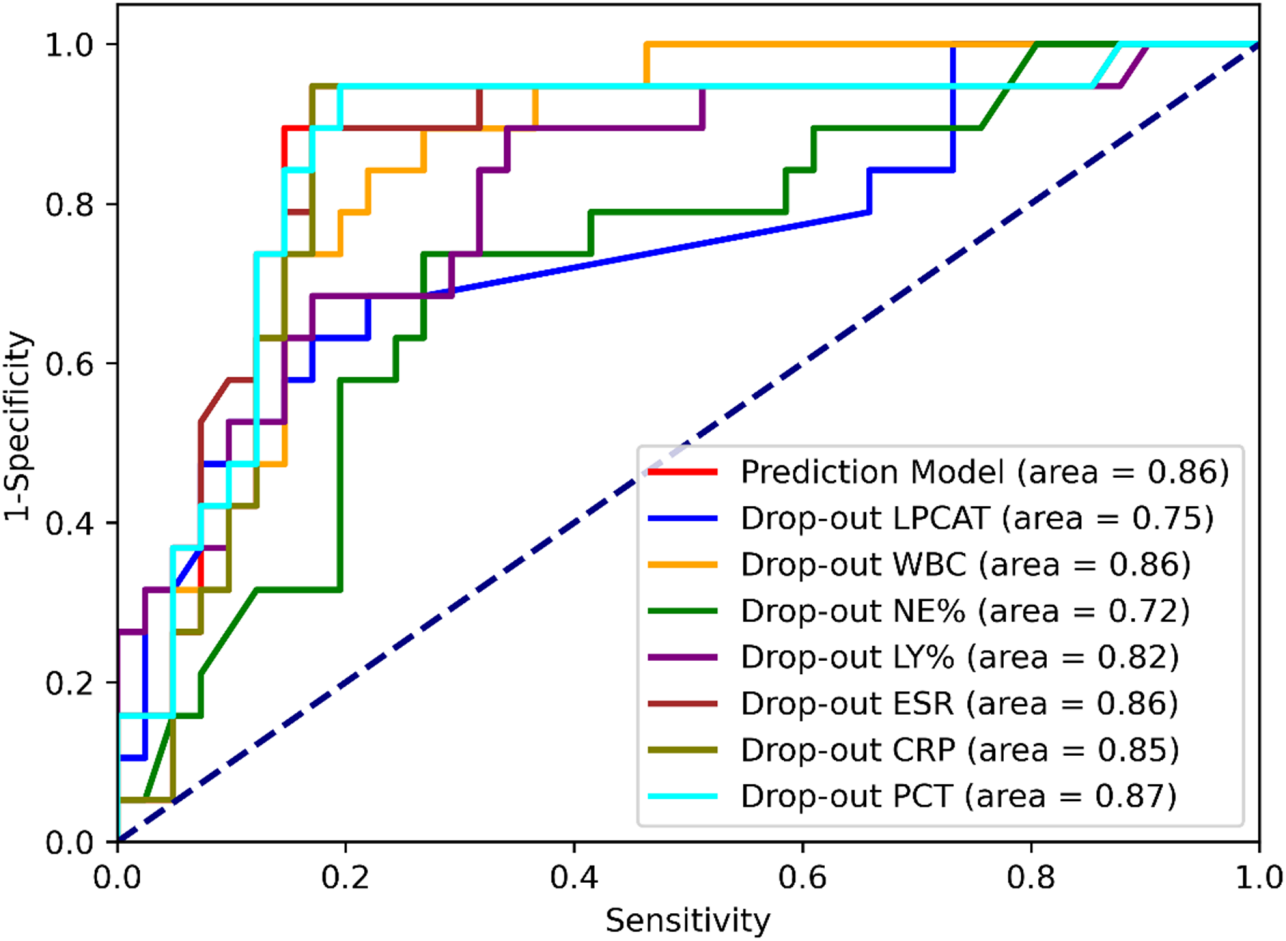
ROC curves of the SCAP prediction model with different dataset inputs.

The performance of the SCAP prediction model was tested on the test dataset, and its sensitivity and specificity were 94.74% and 80.49%, respectively, with an overall accuracy of 85%. For model the AUC of ROC was 0.86. The drop-out method was applied to identify variables with significant contributions (**Table 4** and **Figure 5**). Compared with the original test dataset, the indicators of model performance (including the Youden Index and AUCs) with the dropout datasets of LPCAT and NE% were significantly degraded.

**Table 4:** Performance of the SCAP prediction model with different inputs.

Test Dataset	AUC	Se	Sp	Youden Index
Prediction Model	0.86	0.95	0.80	0.75
Drop-out LPCAT	0.75	0.21	0.98	0.19
Drop-out WBC	0.86	0.95	0.54	0.48
Drop-out NE%	0.72	0.79	0.46	0.25
Drop-out LY%	0.82	0.68	0.76	0.44
Drop-out ESR	0.86	0.89	0.71	0.60
Drop-out CRP	0.85	0.95	0.78	0.73
Drop-out PCT	0.87	0.89	0.80	0.70

Se, sensitivity; Sp, Specificity.

### Value of LPCAT levels in CAP prognosis

The plasma LPCAT level was also the best indicator among the blood test results (**Table 5**); its predictive value was higher than that of the CURB-65 scoring system but lower than that of the PSI, with an optimal cut-off 86.42 ng/ml and an AUC 0.845. The predictive value of the 30-day mortality after combining LPCAT level was improved further along with PSI scores or the CURB-65, with AUCs of 0.902 vs. 0.819 and 0.936 vs. 0.868, respectively.

**Table 5:** Areas under the curve (AUCs) and thresholds for predicting 30-day mortality in patients with CAP.

	AUC	95% CI		Sensitivity	Specificity	Threshold	*p* value
		Lower limit	Higher limit				
LPCAT	0.845	0.798	0.884	80.00%	82.08%	>86.42	<0.0001
WBC	0.747	0.692	0.796	60.00%	88.76%	>15.21	0.0005
NEU %	0.696	0.631	0.756	87.50%	60.39%	> 80.51	0.0071
LYM %	0.775	0.712	0.830	81.25%	67.02%	≤ 9.60	<0.0001
NLR	0.770	0.707	0.826	87.50%	60.73%	>7.07	0.0001
ESR	0.645	0.564	0.720	88.89%	42.74%	>35	0.0566
CRP	0.553	0.470	0.633	45.45%	77.86%	> 139.50	0.5977
PCT	0.730	0.624	0.820	72.73%	77.33%	> 1.33	0.0199
CURB-65	0.819	0.769	0.862	70.00%	81.58%	> 1	<0.0001
PSI	0.868	0.823	0.905	90.00%	85.71%	>109	<0.0001
CURB-65 +LPCAT	0.902	0.862	0.934	90.00%	78.57%	–	<0.0001
PSI+LPCAT	0.936	0.901	0.961	95.00%	79.32%	–	<0.0001

**Figure 6:**
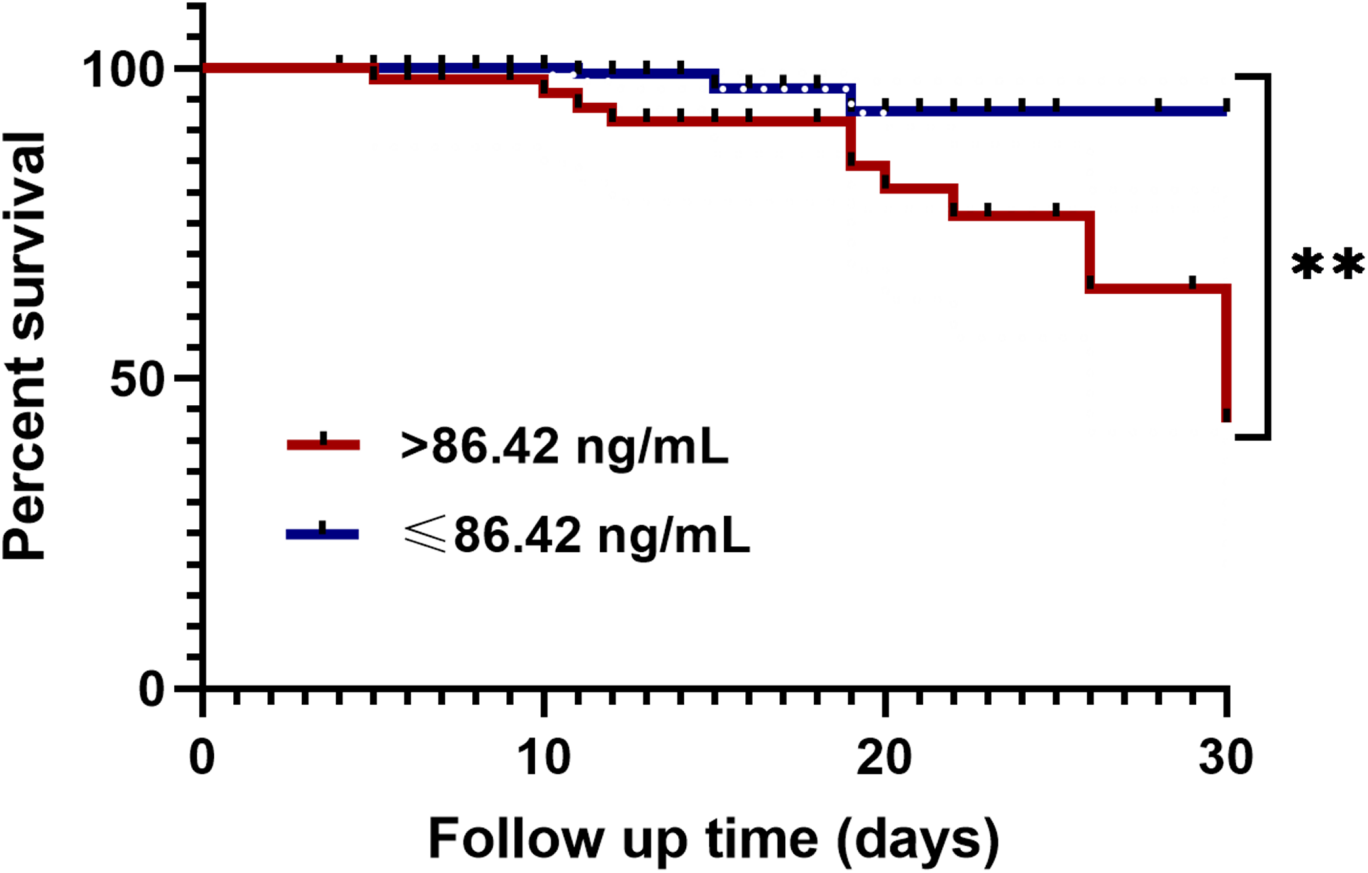
Kaplan-Meier analysis of 30-day mortality in CAP patients with high plasma LPCAT level (> 86.42 ng/ml) and low plasma LPCAT level (≤ 86.42 ng/ml). ** p<0.01.

All CAP presented patients were divided into a high LPCAT group (plasma LPCAT > 86.42 ng/mL) and a low LPCAT group (plasma LPCAT ≤ 86.42 ng/mL) according to the ROC plot of the 30-day mortality prediction shown above. The high LPCAT group showed a significantly higher 30-day mortality rate compared to low LPCAT group (**Figure 6**).

## Discussion

In this research, we illustrated the relationship between plasma LPCAT levels and CAP severity. We measured the plasma LPCAT level among healthy individuals and those patients with different severities of CAP, PSI and CURB-65 scores, and assessed that the increased in plasma LPCAT level was more pronounced in more severe cases than in less severe cases. Moreover, LPCAT level were not influenced by the different pathogens. Finally, we evaluated the value of plasma LPCAT levels in predicting SCAP and 30-day mortality, the LPCAT level was more effective than any of the other blood test results. The predictive values of LPCAT combined with the CURB-65 and PSI scores or other blood test results for SCAP and 30-day mortality were further improved in both the traditional combined diagnostic tests and deep learning models. Taken together, these results show that plasma LPCAT levels can predict the severity of CAP.

LPCAT plays an important role in lipid metabolism and is broadly associated with membrane metabolism and maintenance. LPCAT is upregulated in several carcinomas and is associated with poor prognosis (26), cancer metastasis (27) and proliferation. In patients with colorectal (28) and renal (29) carcinomas, LPCAT expression was enhanced. Previous studies also suggested LPCAT’s involvement in the synthesis of phospholipids in alveolar surfactants (30). Purandare et al. revealed that LPCAT protein was expressed from the initiation of embryogenesis and regulated by oxygen tension, several mitochondrial regulators, and antenatal corticosteroids (31). The role of LPCAT in regulating surfactant phospholipids and respiratory function was also verified in a mouse model (32). LPCAT deficiency can also lead to alveolar epithelial cell apoptosis and promote pulmonary emphysema (33).

However, studies of LPCAT in infectious diseases are limited. An earlier study highlighted the effect of LPCAT for the inflammatory responses of macrophages to lipopolysaccharide and other bacterial stimuli. (34–37). In our previous research, we discovered that a high concentration of phosphatidylcholine correlated with the severity of CAP (38). This may be because LPCAT, which is important for the acylation of LPC to PC, was also significantly higher in SCAP patients, especially in patients who died within 30 days. The LPCAT levels in diseases with different causative pathogens were not significantly different, suggesting that LPCAT is a general indicator that has neither clinical value in distinguishing bacterial, viral, nor other infections.

The correlation between the LPCAT level and the CAP severity score system was also strong in our study. In addition, LPCAT levels were broadly correlated with many clinical and laboratory test results, including respiratory rate, NE%, LY%, NLR, PCT, and PaO_2_. The parameters NE%, respiratory rate, and PaO_2_ are commonly used to evaluate the severity of lung infection, whereas NLR is a convenient biomarker associated with the prognosis and mortality of inflammation (39), tumors (40), and heart failure (41). These results suggest that plasma LPCAT level is a strong indicator of CAP severity.

In our study, the diagnostic LPCAT value was significantly higher than CURB-65 score, PSI score, and other clinical or laboratory indicators. The AUC of LPCAT for predicting SCAP was as high as 0.85, with 84.54% sensitivity and 72.77% specificity. In 30-day mortality prediction, the LPCAT level (AUC=0.845) also performed better than the other laboratory test results, and even better than the CURB-65 score (AUC=0.819) alone, but less efficient than the PSI score (AUC=0.868). By combining PSI or CURB-65 scores along LPCAT, new 30-day mortality assessment methods can be further improved, achieving over 90% sensitivity and 78% specificity. The survival time of CAP patients with plasma LPCAT levels >86.42 ng/mL at admission was significantly shorter than that of CAP patients showing LPCAT< 86.42 ng/mL level, as shown by Kaplan-Meier survival curves.

We attempted to develop a deep-learning-based binary classification (SCAP or NSCAP) model to process plasma LPCAT and other laboratory test results. After viewing multiple machine learning model structures, we chose the MLP as the basic model structure instead of traditional machine learning methods, which includes linear regression, a random forest plot and support vector machine. The MLP model input is more tolerant because it requires less feature engineering. The SCAP prediction model achieved 94.74% sensitivity and 80.49% specificity, with an overall accuracy of 85.00%, which had the highest sensitivity and the second highest specificity. After applying the drop-out method, LPCAT level and NE% seemed to have the highest weights in the SCAP prediction model, as the performance of the model was significantly degraded with the drop-out datasets of LPCAT and NE%. The LPCAT and the indicators of peripheral blood cell count missing in prediction variables significantly reduced the Youden index and AUCs, whereas the Youden index and AUCs of inflammatory biomarker (including erythrocyte sedimentation rate, CRP, and PCT) drop-out datasets were like those of the complete dataset prediction. The lower importance of erythrocyte sedimentation rate, CRP, and PCT in distinguishing SCAP from NSCAP is inconsistent with previous research findings (42–44). A possible explanation is that the missing values of these inflammatory biomarkers were greater than those of the other laboratory test results. Therefore, the weights of inflammatory biomarkers were lower in the prediction model. However, our model was trained to consider missing input values because we applied L2-norm regularization to limit the weight for each input variable. The ability to consider missing input variables made the prediction model much more tolerant and robust. Taken together, plasma LPCAT levels can improve prognosis prediction and contribute to improved clinical practice.

However, the process of increasing LPCAT levels are elevated in severe cases still remains unclear. LPCAT is an essential component of the Lands cycle and participates in lipid metabolism and membrane maintenance in multiple types of cells and tissues (12). Combined with our previous study on lipid profiles (37) and another lipid metabolism enzyme, LPEAT (37), in patients with SCAP, we speculated that the elevation of LPCAT may be related to lung tissue destruction in SCAP because LPCAT is abundantly expressed in type II alveolar epithelial cells (8).

Our study had certain limitations. First, we only collected blood samples at admission; therefore, we could not elucidate the trends in LPCAT levels in patients with CAP. The dynamic trend of LPCAT concentration changes in the progression of CAP inflammation and the response to treatment require further follow-up. Future studies should assess the long-term prognostic value of LPCAT. Moreover, although our study showed that LPCAT levels are not related to specific pathogens, future studies should focus on changes in LPCAT levels during different pathogeneses.

## Conclusion

The plasma LPCAT level at the time of admission were significantly higher in SCAP patients and strongly predicted SCAP in CAP patients but with no correlation to etiology. Combining LPCAT with available CAP severity scores (CURB-65 or PSI) can further improve the 30-day mortality prediction.

## Data availability statement

The original contributions presented in the study are included in the article/**Supplementary Material**. Further inquiries can be directed to the corresponding authors.

## Ethics statement

This study was performed in line with the principles of the Declaration of Helsinki. This study was approved by the Institutional Review Board of the Peking University People’s Hospital (2016PHB202-01). The studies were conducted in accordance with the local legislation and institutional requirements. The participants provided their written informed consent to participate in this study. Written informed consent was obtained from the individual(s) for the publication of any potentially identifiable images or data included in this article.

## Author contributions

LC: Writing – original draft, Writing – review & editing. JX: Writing – review & editing. LZ: Data curation, Investigation, Writing – review & editing. YH: Investigation, Software, Writing – review & editing. SF: Data curation, Investigation, Methodology, Software, Writing – review & editing. XM: Writing – review & editing. WY: Investigation, Methodology, Software, Writing – review & editing. YT: Funding acquisition, Project administration, Writing – review & editing. YW: Formal analysis, Funding acquisition, Writing – review & editing. ZG: Conceptualization, Formal analysis, Investigation, Methodology, Project administration, Resources, Supervision, Writing – review & editing.

## Supplementary material

SUPPLEMENTARY DATA SHEET 1 Detailed parameters for the dataset created.
