# Supplementary material for: Lysophosphatidylcholine acyltransferase level predicts the severity and prognosis of patients with community-acquired pneumonia: a prospective multicenter study
Source: Front Immunol. 2024 Jan 8;14:1295353. doi: 10.3389/fimmu.2023.1295353 (PMC10800399; doi:10.3389/fimmu.2023.1295353)
Supplement: SUPPLEMENTARY DATA SHEET 1 — Detailed parameters for the dataset created. [file DataSheet_1.docx]

We created a dataset with the following features and labels: 1) features were eight laboratory test results, including LPCAT level, white blood cell (WBC) count, neutrophil percentage (NE%), neutrophil/lymphocyte ratio (NLR), lymphocyte percentage (LY%), procalcitonin, C-reactive protein (CRP) and erythrocyte sedimentation rate (ESR); 2) labels were the binary variable of “1” representing SCAP and “0” representing NSCAP. Missing values in features were assigned −1 to avoid a system error. To train and test the performance of the SCAP prediction model, we split the entire dataset into a training and test datasets at a ratio of 4:1 but maintained the proportion of patients with SCAP and CAP at the same level using the Python package scikit-learn (v1.1.2). We also applied 5-fold cross-validation to the training dataset to adjust the hyperparameters. The dropout method was applied to further investigate the contribution of each variable in the data set to the prediction model. We developed seven drop-out datasets based on the original test dataset corresponding to seven feature indicators except for NLR (a missing value for either NE% or LY% will result in the absence of NLR). In each dropout dataset, we assigned the value of −1 (representing the missing value) to all patients’ corresponding indicators. In the NE% drop-out and LY% drop-out datasets, we assigned all variable values of −1 to both NE% (LY%) and NLR.
